# A polygenic risk score contributes to identifying individuals with the potential for cognitive function improvement

**DOI:** 10.1002/alz.086348

**Published:** 2025-01-03

**Authors:** Daichi Shigemizu, Kosuke Fujita, Shumpei Niida, Kouichi Ozaki, Takashi Sakurai, Hidenori Arai

**Affiliations:** ^1^ National Center for Geriatrics and Gerontology, Obu, Aichi Japan; ^2^ RIKEN Center for Integrative Medical Sciences, Yokohama, Kanagawa Japan; ^3^ Hiroshima University Graduate School of Biomedical and Health Sciences, Hiroshima, Hiroshima Japan

## Abstract

**Background:**

A polygenic risk score (PRS) estimates an individual’s genetic liability for diseases based on accumulated genetic variations. While the PRS has been shown to contribute to the prediction of dementia risk, its utility in predicting the effectiveness of multifactorial interventions to reduce the risk of dementia remains unclear.

**Methods:**

A genome‐wide association study (GWAS) was conducted to identify dementia‐related traits in the Japanese population. Subsequently, PRSs were constructed based on GWAS results using PRSice‐2 software. The Japan‐multimodal intervention trial for prevention of dementia (J‐MINT) is an RCT to assess the effect of multi‐domain interventions for subjects with mild cognitive impairment. The J‐MINT samples were classified into high‐risk and low‐risk groups using the Japanese PRS. Within each risk group, cognitive function improvement due to the intervention was assessed using Fisher’s exact test.

**Results:**

We conducted a GWAS with 3962 AD cases and 4074 controls for 4,578,811 genetic markers that passed stringent quality control filters. The optimal PRS, composed of 12,818 SNPs and *APOE* status, was developed at a p‐value threshold of 0.090, selected for its highest model fit with an R^2^ score of 0.023. Subsequently, the PRS classified 289 J‐MINT samples into 99 high‐risk and 190 low‐risk groups. Individuals in the young‐old group (aged <75 years) showed significant cognitive improvement through the intervention in the high‐risk group; however, those in the old‐old group (aged 75 years) did not demonstrate such potential in either risk group.

**Conclusions:**

While we have successfully developed a Japanese PRS with high predictive ability, it is important to note that our dataset is limited and relatively small. To achieve an accurate AD PRS for the Japanese population, further validation studies are necessary, involving a larger dataset or conducting a meta‐analysis with another Japanese cohort. Additionally, our study highlights the potential utility of the PRS in screening subjects for cognitive improvement, especially for individuals with high PRS in the young‐old group.

